# GWO-Based Multi-Stage Algorithm for PMDC Motor Parameter Estimation

**DOI:** 10.3390/s23115047

**Published:** 2023-05-24

**Authors:** Adam Pawlowski, Maciej Ciezkowski, Slawomir Romaniuk, Zbigniew Kulesza

**Affiliations:** Department of Automatic Control and Robotics, Faculty of Electrical Engineering, Bialystok University of Technology, ul. Wiejska 45D, 15-351 Bialystok, Poland; m.ciezkowski@pb.edu.pl (M.C.); s.romaniuk@pb.edu.pl (S.R.); z.kulesza@pb.edu.pl (Z.K.)

**Keywords:** PMDC parameter estimation, PMDC motor model, GWO

## Abstract

During the design of a wheeled mobile robot, the problem of the proper selection of the parameters of its motor controllers was encountered. Knowing the parameters of the robot’s Permanent Magnet Direct Current (PMDC) motors, precise tuning of the controllers can be performed, which then results in improved robot dynamics. Among many methods of parametric model identification, optimization-based techniques, particularly genetic algorithms, have gained more and more interest recently. The articles on this topic present the results of parameter identification, but they do not refer to the search ranges for individual parameters. With too wide a range, genetic algorithms do not find solutions or are time-inefficient. This article introduces a method for determining the parameters of a PMDC motor. The proposed method performs an initial estimation of the range of searched parameters to shorten the estimation time of the bioinspired optimization algorithm.

## 1. Introduction

PMDC motors are widely utilized, e.g., as drive units of wheeled mobile robots, or as DC–DC converters [1]. High-speed accurate performance of a robot can be provided by setting the proper parameters of the motor speed/torque controllers. The most frequently used controllers are digital PID controllers [2], and their modifications [3]. Other algorithms for PMDC control can also be found. The authors in [4] introduced the model reference adaptive system (MRAS)-based method to control the motor, and compared the results with a PI controller. Different simulation methods can be used to design the controller of the PMDC motor, yet the parameters of the motor model need to be known in advance [5]. In the literature, a simple linear viscous friction model is mostly implemented. The friction model is more complex and takes into account Coulomb, Stribeck, and sticky friction as presented by the authors in paper [6]. The shape of the friction characteristics is a nonlinear curve and is often approximated by an exponential or polynomial function, as demonstrated by the authors in paper [7].

There are many methods of parametric model identification [8]. The most popular of the optimization methods used for identification is the Recursive Least-Squares (RLS) method [9]. It is recommended for its simplicity and applicability to real systems in addition to its advantage of lower computational complexity. An interesting solution for PMDC model estimation using binary-valued measurements is presented in [10]. In the paper, the suitable regression model is used to model the relationship between the input voltage and the output speed of the motor, to support online estimation utilizing cheap and low-complex binary sensors. A similar approach was utilized by the authors in [5,11], where the authors presented a deeper description and a wider scope of examples. A sensorless method for speed estimation of a PMDC motor is shown in [12]. The authors utilized the RLS method and the first-order mathematical model of the PMDC to reduce the number of samples required for online implementation. The authors in [13] presented a method for estimating a PMDC motor speed considering the effect of the armature teeth-slots and commutation. Different motor responses are used to gather the data required for parameter estimation. In [14], authors used a Pseudo-Random Binary Sequence (PRBS) for the system identification process. The authors in [15] utilized multiparametric programming to estimate the parameters of the DC motor. Multiparametric programming is a mathematical optimization technique that can be used to solve parameter estimation problems. In this method, the parameters of a system are estimated by minimizing a cost function subject to a set of constraints. The authors in paper [16] suggested a novel approach for parameter estimation based on the Rao-1 algorithm, which is a modified version of the Kalman filter. Another approach to motor parameter estimation is proposed in [17]. The article presents the method for identifying the parameters of a DC motor using a compound least-squares (CLS) approach. Articles [18,19] propose novel methods for identifying the parameters of a DC motor based on algebraic equations. Motor parameter estimation methods utilizing Swarm Intelligence algorithms are presented in [20,21].

Optimization-based methods, in particular the techniques based on genetic algorithms, have gained broad interest [22]. Particularly interesting is the work presented by the authors in [23], which uses the Gray Wolf Optimization (GWO) genetic algorithm to determine the parameters of a brushed electric motor. However, the downside of the method is a very wide range of searched parameters, which results in a long estimation period and uncertainty of the obtained results. Furthermore, the fitness/cost function presented by the authors can be questioned due to the lack of weighting for current and speed components. Due to the significant order-of-magnitude difference between the current and speed, the above-mentioned fitness/cost function focuses on matching the speed waveform, almost completely ignoring the current response of the motor.

All the above-mentioned methods can be characterized in terms of the accuracy, complexity, and time of parametric model identification. The time of identifying the parameters is directly related to the range in which the solution is sought. To increase the efficiency of optimization methods, it is necessary to roughly define the values of the parameters, to establish search ranges for individual parameters.

In the following paper, a new method for quick estimation of the PMDC motor parameters is proposed. The method consists of two stages. In the former one, rough values of parameters are calculated, while in the latter one, they are carefully evaluated to estimate the fine values. Since the second stage requires a little more time to finish, the method intends to shorten the whole procedure by starting fine parameter estimation with rough estimates of the values from the first stage.

## 2. Methods

This section presents the methods of modeling and estimating PMDC motor parameters.

### 2.1. Modeling of the PMDC Motor

**Figure 1:**
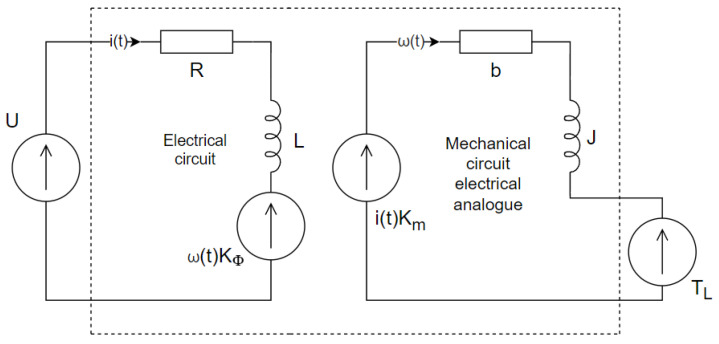
Electro-mechanical model of the PMDC motor; where ω is the angular speed of the motor shaft, *i* is the current flowing through the rotor winding, and *u* is the supply voltage.

The principle of operation of a PMDC motor is related to basic electrical and mechanical laws [24]. The flow of current through the rotor winding creates a force as a result of the interaction of the stator’s magnetic field with the rotor’s magnetic field. The torque of the force acting on the rotor is proportional to the current flowing through the rotor winding, and the mechanical constant $K_{m}$. A DC motor with a permanent magnet can be modeled in the form of two analogous circuits: electrical and mechanical (see Figure 1). The parameters of the electric circuit are rotor winding resistance *R*, rotor winding inductance *L*, and electromotive constant $K_{\phi }$. The parameters of the mechanical circuit are friction coefficient *b*, rotor inertia *J*, mechanical constant $K_{m}$, and loading torque $T_{L}$. Due to the analogy between the two circuits, the mechanical circuit is modeled using electrical symbols.

The mathematical model of the motor can be described by two differential equations. Using the second Kirchhoff law, the following relationships can be obtained:

(1) $$u\left(t\right)=i\left(t\right)R+\frac{di(t)}{dt}L+\omega \left(t\right)K_{\phi }$$

(2) $$K_{m}i\left(t\right)=\frac{d\omega (t)}{dt}J+\omega \left(t\right)b+T_{L}$$

The total power of the motor (or any other electrical device) is expressed as P=u(t)i(t). According to Equation (1), the total power of the motor is:

(3) $$P=u\left(t\right)i\left(t\right)=\left(i\left(t\right)R+\frac{di(t)}{dt}L+\omega K_{\phi }\right)i\left(t\right)=i{\left(t\right)}^{2}R+i\left(t\right)\frac{di(t)}{dt}L+\omega K_{\phi }i(t)$$

The motor power that is converted into mechanical power is described by the third term of Equation (3) (the first term describes the power associated with Joule–Lenz heat, while the second describes the magnetization of the coil). Since the mechanical power of a rotating motor is also defined as $P_{m}=\omega K_{m}i\left(t\right)$, it can be assumed that in all the previous and following Equations, there is an equality:

(4) $$K_{\phi }=K_{m}=K$$

Equations (1) and (2) form a system of differential equations, where the unknown functions are i(t) and ω(t). The most common approach to solve this differential equations system is to use numerical methods (e.g., using Simulink). However, this system can be solved analytically. The advantage of such an approach is that it speeds up further calculations performed during numerical optimization. Analytical solutions of Equations (1) and (2) take the form:

(5) $$\omega \left(t\right)=\frac{KUe^{-\frac{t(A+bL+JR)}{2JL}}\left(A\left(-2e^{\frac{t(A+bL+JR)}{2JL}}+e^{\frac{At}{JL}}+1\right)+\left(e^{\frac{At}{JL}}-1\right)\left(bL+JR\right)\right)}{2A\left(bR+K^{2}\right)}$$

(6) $$i\left(t\right)=\frac{Ue^{-Bt}\left(\left(e^{\frac{At}{JL}}-1\right)\left(b^{2}\left(-L\right)+bJR+2JK^{2}\right)-Ab\left(e^{\frac{At}{JL}}-2e^{Bt}+1\right)\right)}{2A\left(bR+K^{2}\right)}$$

where $A=\sqrt{b^{2}L^{2}+J^{2}R^{2}-2JL\left(2K^{2}+bR\right)}$ and $B=\frac{A+bL+JR}{2JL}$. These solutions are obtained under the following assumptions: u(t)=U=const, i(0)=ω(0)=0.

### 2.2. Characteristic Points Method

To simplify and speed up calculations, the characteristic points method (CPM) of parametric model identification is introduced. The method assumes that the values of model parameters are calculated based on current and speed values at characteristic points for which the derivative is equal to zero.

**Figure 2:**
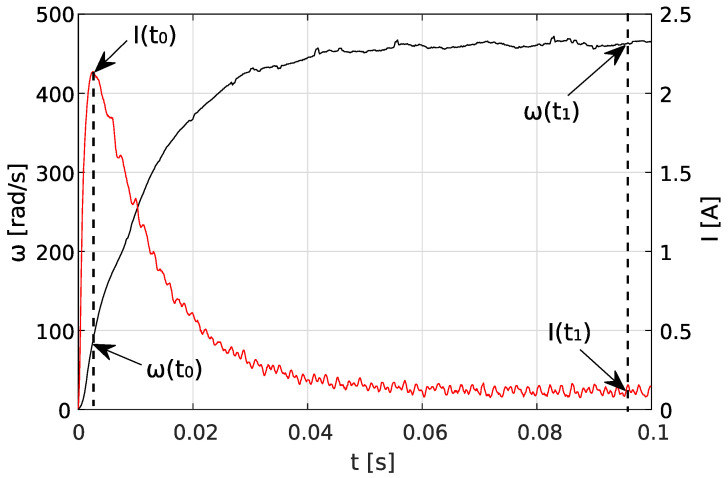
Characteristic points of current (red) and angular speed (black) step responses.

Equation (1) for characteristic points $t_{0}$ and $t_{1}$ (see Figure 2) can be written as:

(7) $$u\left(t_{0}\right)=i\left(t_{0}\right)R+\omega \left(t_{0}\right)K$$

(8) $$u\left(t_{1}\right)=i\left(t_{1}\right)R+\omega \left(t_{1}\right)K$$

Equation (2) in time $t_{1}$, on the other hand, can be written in the following form:

(9) $$Ki\left(t_{1}\right)=\omega \left(t_{1}\right)b+T_{L}$$

Based on Equations (7)–(9) with condition $u\left(t_{0}\right)=u\left(t_{1}\right)=U$, the following parameters of the PMDC motor model can be obtained:

(10) $$R=\frac{u(\omega \left(t_{1}\right)-\omega \left(t_{0}\right))}{i\left(t_{0}\right)\omega \left(t_{1}\right)-i\left(t_{1}\right)\omega \left(t_{0}\right)}$$

(11) $$K=\frac{U-i(t_{1})R}{\omega (t_{1})}$$

(12) $$b=\frac{i\left(t_{1}\right)K-T_{L}}{\omega (t_{1})}$$

(13) $$L=\tau_{e}R$$

(14) $$J=\frac{\tau_{m}K^{2}}{R}$$

where $\tau_{m}$ and $\tau_{e}$ are mechanical and electrical time constants, respectively. Observing a certain dependence for most DC motors that $\tau_{e}$ <<$\tau_{m}$, we can simply assume that it is a first-order system. We can approximately assume that moments after forcing the voltage on the motor terminals, the system behaves like a regular RL circuit. Therefore, the electrical time constant can be estimated as the time it takes for the current to reach 63.2% of its maximum value. The error in this estimation will increase as the $\tau_{m}/\tau_{e}$ ratio increases. Due to the fact that the CPM method is used only to determine the ranges, such a simplification is justified.

It should be noted that the described CPM method is not precise, because it is strongly sensitive to unavoidable measurement noise at characteristic points $t_{0}$ and $t_{1}$ and an error in the graphical determination of $T_{e}$ and $T_{m}$. However, it can roughly estimate the parameter values and determine the scope of their search for the optimization method.

### 2.3. Optimization Method

The purpose of the below analysis is to find a nonlinear fit between experimental and theoretical data using an optimization method. The experimental data are the angular speed $\hat{\omega }\left(t_{i}\right)$ and current $\hat{i}\left(t_{i}\right)$ of the motor (for example, such as those shown in Figure 2), while the theoretical data are the results $\omega (t_{i})$ and current $i(t_{i})$ obtained from Equations (5) and (6), which depend on motor parameters *R*, *K*, *b*, *J*, and *L*.

The goal of the optimization algorithm is to find such *R*, *K*, *b*, *J*, *L* that minimize the following objective function:

(15) $$F\left(R,K,b,J,L\right)=\frac{\sum_{i=1}^{N}\left(a\left(\omega \left(t_{i}\right)-\hat{\omega }\left(t_{i}\right)\right)\right){}^{2}}{N}+\frac{\sum_{i=1}^{N}\left(i\left(t_{i}\right)-\hat{i}\left(t_{i}\right)\right){}^{2}}{N}$$

where *i* is the sample number and *N* is the number of step response samples measured in the experiment.

Thus, the minimization problem can be presented in the following form:

(16) $$\begin{array}{c} \left(R,K,b,J,L\right)=\underset{(R,K,b,J,L)\in R}{\arg\min}\;\;F(R,K,b,J,L) \end{array}$$

To normalize the values of motor speed and current, a weighting factor in Equation (15) is introduced. This coefficient takes the form:

(17) $$a=\frac{\sqrt{\sum_{i=1}^{N}\hat{i}\left(t_{i}\right)}}{\sqrt{\sum_{i=1}^{N}\hat{\omega }\left(t_{i}\right)}}$$

Equation (15) clearly shows the advantage of analytical solutions of differential Equations (1) and (2)—the values of $\omega (t_{i})$ and $i(t_{i})$ can be calculated immediately from Equations (5) and (6) instead of numerical simulation.

**Figure 3:**
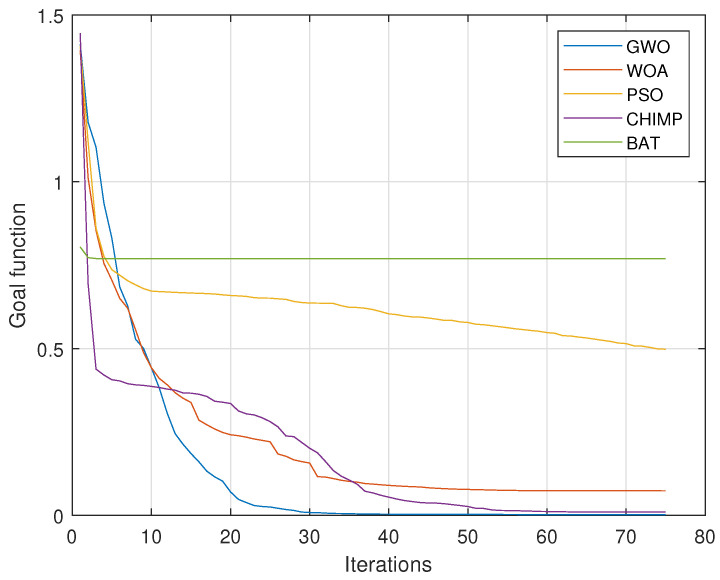
Comparison of different optimization algorithms for the test data.

Optimization algorithms search the solution space in different ways. Some are better suited to given issues, and some are worse. Optimization methods differ in effectiveness and time of finding solutions. To select the best bioinspired algorithm to solve the presented problem, preliminary simulation tests were performed. A set of test data was prepared, and parameters were identified using five different algorithms. The value of the objective function and the number of iterations served as a qualitative indicator for each of the algorithms. Algorithms that were selected for the comparison group were GWO algorithm [25], whale optimization algorithm (WOA) [26], particle swarm optimization (PSO) algorithm [27], chimp optimization algorithm (CHIMP) [28], and bat algorithm (BAT) [29]. The obtained results are presented in Figure 3.

In solving the presented identification problem, the GWO algorithm turned out to be the most effective, which is why it was chosen.

GWO is a metaheuristic algorithm inspired by the hunting behavior of gray wolves. The algorithm mimics the social hierarchy and hunting mechanism of gray wolves to solve optimization problems.

**Figure 4:**
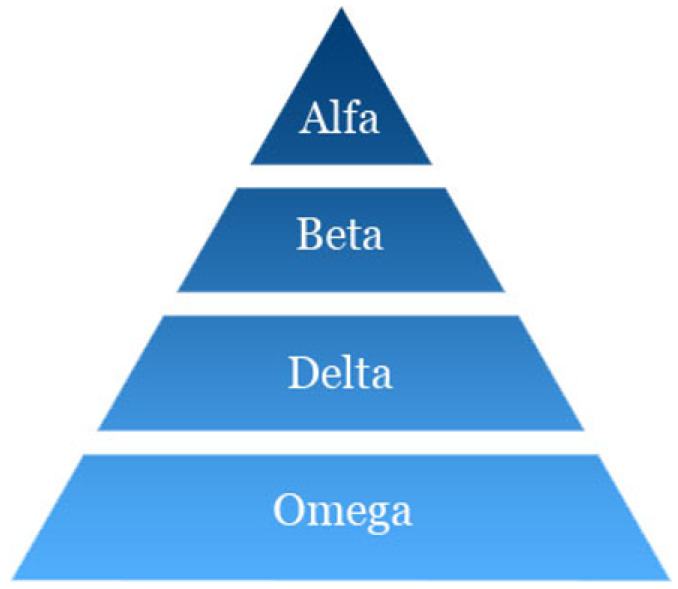
Hierarchy found in a pack of gray wolves.

The algorithm starts with the initialization of a population of candidate solutions, which are called wolves. The position of each wolf in the search space represents a potential solution to the problem. The population of wolves is then divided into four groups, namely alpha, beta, delta, and omega wolves Figure 4, based on their fitness values. The alpha wolf has the best fitness value, while the omega wolf has the worst fitness value.

In each iteration of the algorithm, the position of each wolf is updated based on its position and the positions of the alpha, beta, and delta wolves. This update is performed using three different equations, each representing one of the three types of wolf behavior: searching, encircling, and attacking. The search behavior is used by the omega wolf to explore the search space, while the encircling and attacking behaviors are used by the alpha, beta, and delta wolves to converge toward the optimal solution.

The algorithm terminates when a stopping criterion is met, such as a maximum number of iterations or a desired level of fitness is reached. The final solution is the position of the alpha wolf, which represents the best solution found by the algorithm.

Overall, the Gray Wolf Optimizer is a simple yet effective algorithm that has shown promising results in solving a wide range of optimization problems, including engineering, finance, and machine learning. Its ability to balance exploration and exploitation makes it a popular choice for solving complex optimization problems.

The following equations are proposed to mathematically model the encircling behavior of gray wolves during the hunt:

(18) $$D=|CX_{p}\left(t\right)-x\left(t\right)|$$

(19) $$X\left(t+1\right)=X_{p}\left(t\right)-AD$$

where $X_{p}$ is the victim position vector, X is the gray wolf position vector, *t* indicates the current iteration, A and D are coefficient vectors. Vectors A and C are computed as follows:

(20) $$\begin{array}{c} A=2ar_{1}-a\\ C=2r_{2} \end{array}$$

where vector a linearly decreases during iteration from 2 to 0, $r_{1}$ and $r_{2}$, are random vectors with a value between 1 and 0.

To mathematically simulate the hunting behavior of gray wolves, it is assumed that the alpha (the best candidate solution), beta, and delta have a better understanding of the potential location of the prey. Thus, the first three best solutions obtained thus far are saved, and the other search agents (including the omegas) are required to update their positions according to the position of the best search agent. In this regard, the following formulas are proposed:

(21) $$D_{\alpha }=\left|C_{1}\cdot X_{\alpha }-X\right|,D_{\beta }=\left|C_{2}\cdot X_{\beta }-\vec{X}\right|,D_{\delta }=\left|C_{3}\cdot X_{\delta }-X\right|$$

(22) $$X_{1}=X_{\alpha }-A_{1}\cdot \left(D_{\alpha }\right),X_{2}=X_{\beta }-A_{2}\cdot \left(D_{\beta }\right),X_{3}=X_{\delta }-A_{3}\cdot \left(D_{\delta }\right)$$

(23) $$X\left(t+1\right)=\frac{X_{1}+X_{2}+X_{3}}{3}$$

A more detailed description of the algorithm can be found in [25].

### 2.4. Combined CPM-GWO Method

A common problem that occurs when solving optimization tasks is the possibility of the objective function becoming stuck in a local minimum. To prevent this, constraints can be imposed on the parameters. This is generally problematic because it requires a priori knowledge of the range of solutions being sought.

To overcome this difficulty, a new method called CPM-GWO is proposed, which combines the two algorithms: the characteristic points method (CPM) described in Section 2.2 and the GWO introduced in Section 2.3. The estimation process in this case is as follows: first, make a rough identification of model parameters using the characteristic points method, then use model parameters *p* obtained during the first stage (i.e., CPM) to define the solutions range according to the following notation:

(24) [0.1p,10p],p∈R

**Figure 5:**
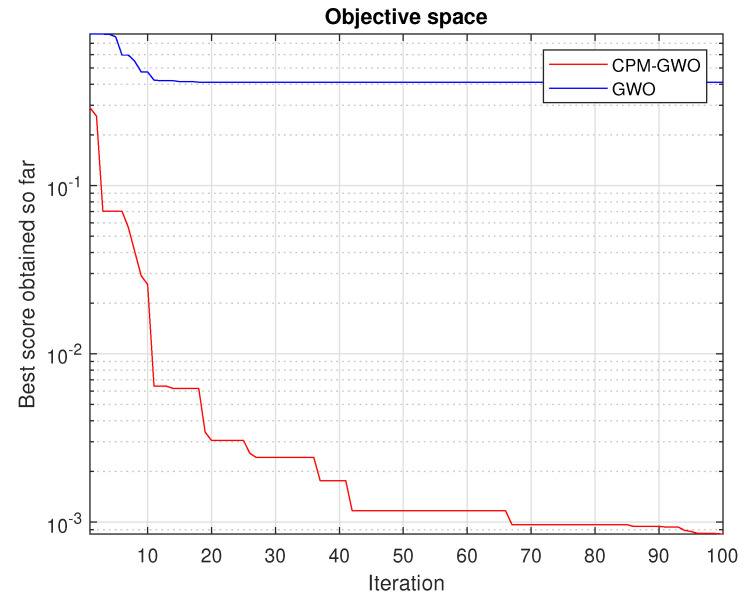
Comparison of the GWO with a too-wide initial range and the CPM-GWO methods.

The range is defined as the area of searching for individual parameters in the optimization method. Figure 5 shows an example comparison of the GWO method with the searching area taken as [0.0000001,10] (which appears to be a reasonable area for the PMDC motor parameters) and the CPM-GWO method where the search area was narrowed using the CPM solution.

It can be seen that despite using the same objective function given by Equation (15), the GWO solution is stuck in the local minimum.

The discrepancy in the GWO results can be seen in detail in Table 1, where the methods used are compared.

## 3. Experimental Results

Model identification was carried out for the Buhler DC motor 31 × 51 1.13.021.764.

Step responses i(t) and ω(t) of the selected motor were measured. Motor parameters were then identified at a nominal voltage of 12 V using CPM, GWO and CPM-GWO methods.

### 3.1. Test Rig

**Figure 6:**
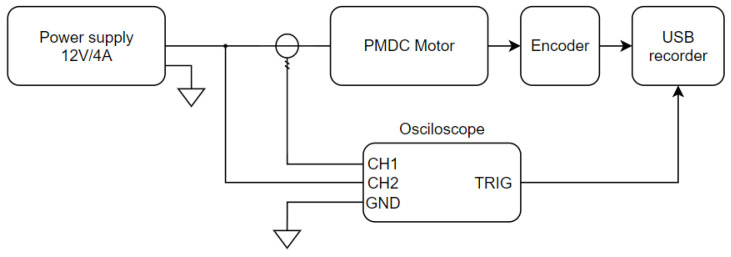
Schematic diagram of the experimental test rig.

**Figure 7:**
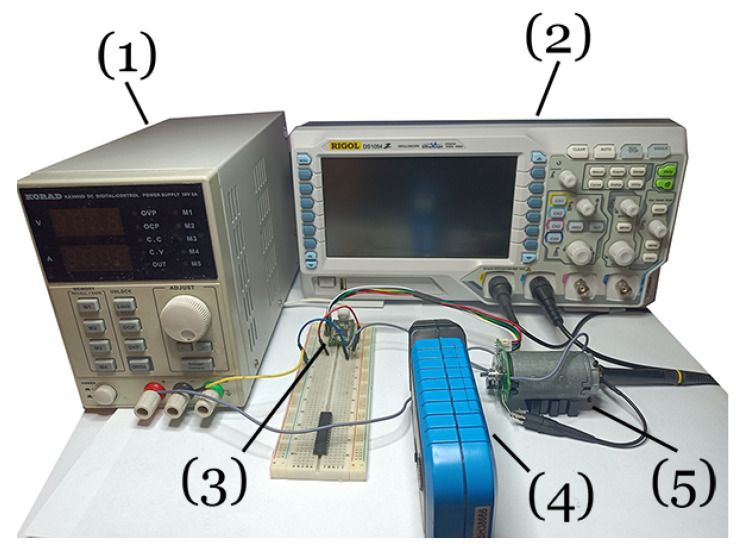
Photo of the experimental test rig.

A block diagram of the test rig is shown in Figure 6 and the photo of the rig in Figure 7. The motor was connected to a supply voltage source KA3005D set for the rated voltage of 12 V (1). An oscilloscope (2) was used to record the voltage and current of the motor (5). A current probe (4) was placed at the power cord to measure the current value. Motor rotational speed was measured using an optical encoder and a USB frequency recorder Teensy 3.2 (3) triggered by an oscilloscope. The measurement of the step response of the motor was forced by switching on the key controlled from the Teensy board.

### 3.2. Parameters Estimation Using the CPM

**Figure 8:**
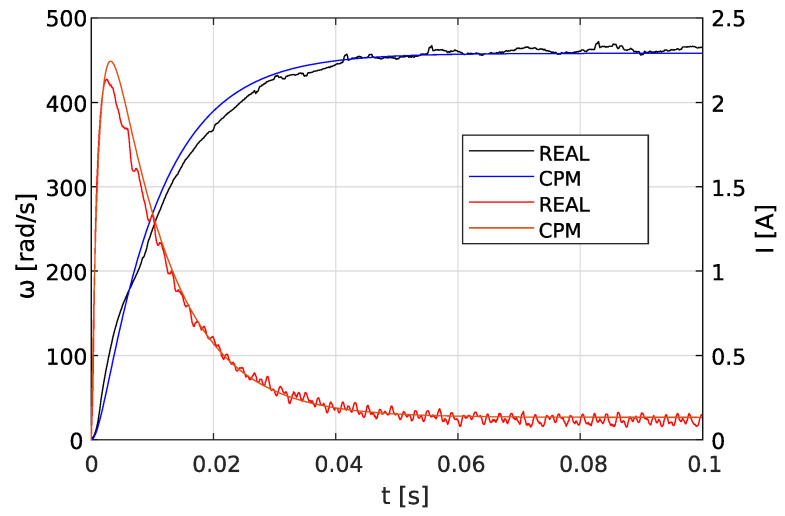
Step response for parameters calculated by the CPM.

Based on the recorded step responses of the Buhler gear motor, the points for which the $\frac{di}{dt}=0$ condition is met were determined. Then, using Equations (10)–(14), parameters *R*, *L*, *K*, *J*, *b* were calculated. In the next stage, using the graphical method, time constants $\tau_{m}$ and $\tau_{e}$ were determined. Next, parameters *J* and *L* were calculated according to Equations (13) and (14). Step responses for model parameters obtained with the proposed characteristic points method were generated and are presented in Figure 8. Parameter values obtained through the characteristic points method are listed in Table 1.

### 3.3. Motor Model Identification Using CPM-GWO

**Figure 9:**
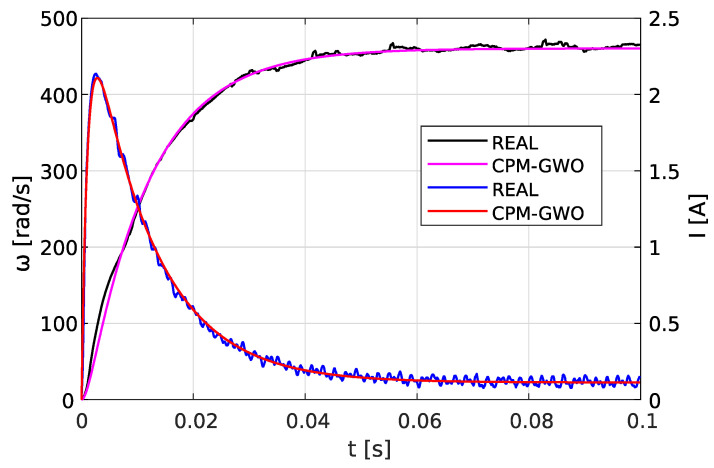
Step response for parameters calculated by the CPM-GWO.

Using the results obtained from the CPM method, search ranges for individual parameters were determined in accordance with Equation (24). The objective function was assumed in the form given by Equation (15). A comparison of experimental and simulation step responses obtained for the identified model parameters is presented in Figure 9.

The obtained values of the PMDC model parameters are collated in Table 1, together with the reference values given by the manufacturer of the tested motor. To improve the overall credibility of the obtained results, parameter estimation was repeated 100 times for each of the CPM-GWO and GWO methods. Table 1 describes the average values of the parameters obtained throughout this process, and the minimum, maximum and standard deviation of the estimated values.

The results shown in Table 1 clearly demonstrate that the GWO method with an a priori assumed searching range [0.0000001, 10] produces unreliable results. The average values of parameters *K*, *J*, *b* are not close to the factory reference values. In addition, these results are characterized by a large scatter, which can be seen in the standard deviation, min and max values. In contrast, it can be seen that the results for the CPM-GWO method are comparable to the reference factory data. According to the data in Table 1, the CPM-GWO method produces a little scatter in the results, as demonstrated by the value of the standard deviation, and in the worst cases (see min and max) the results differ from the average by a few percent at most. The exception is the value of the friction coefficient for which the standard deviation is a percentage point larger (σ = 7.81 ×10${}^{-7}$ to avg = 6.11 ×10${}^{-6}$) compared to the rest of the parameters. The problem with the friction coefficient is discussed further.

**Figure 10:**
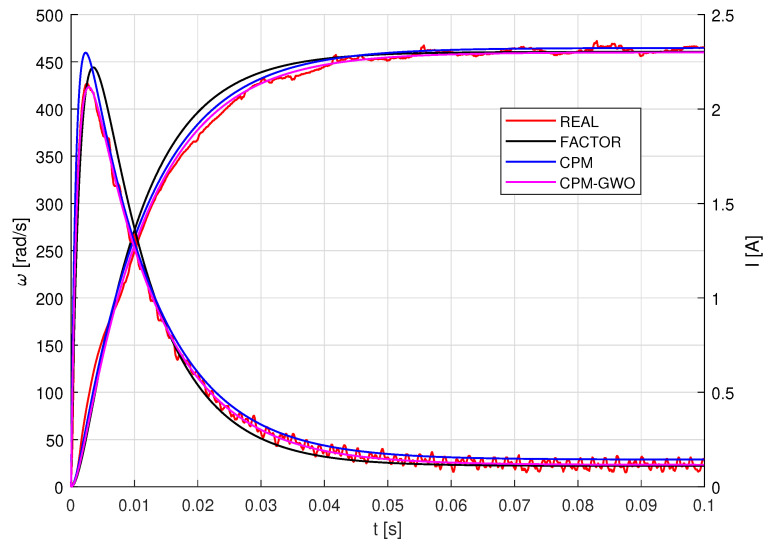
Step responses of the motor model for parameter values obtained by different methods for 12 V nominal voltage.

Step responses of the motor for the calculated parameters and the reference values provided by the manufacturer are shown in Figure 10.

### 3.4. Motor Identification for a Wide Range of Supply Voltages

**Figure 11:**
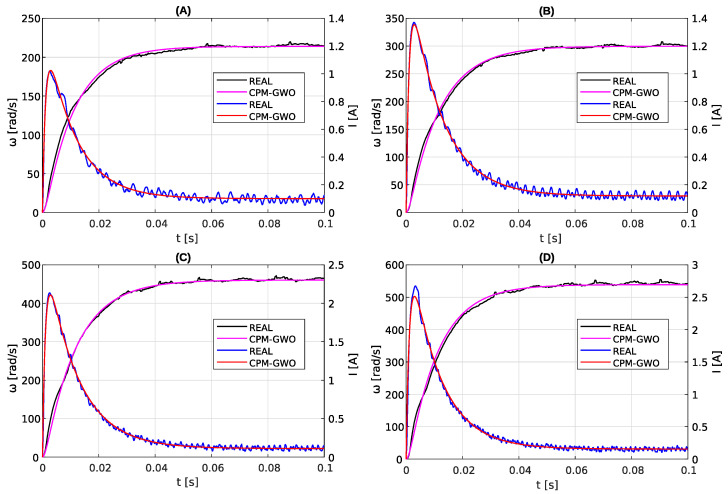
Comparison of the measured step response with model responses of the PMDC motor for parameters estimated by the proposed combined CPM-GWO method for different voltages: 6 V (**A**), 8 V (**B**), 12 V (**C**), 16 V (**D**).

**Figure 12:**
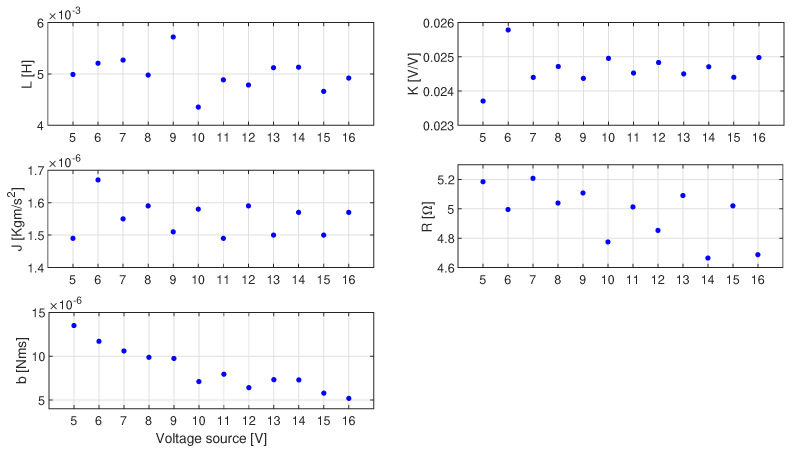
Parameter values estimated by the proposed CPM-GWO method for the Buhler DC motor 31 × 51 1.13.021.764 for various voltages.

The identification of motor parameters described in Section 3.2 and Section 3.3 was performed for a nominal voltage of 12 V. Thus, the results obtained can be compared with the parameters declared by the motor manufacturer. However, for purposes such as simulation, it is required to know the parameters of the motor for any voltage and not just the nominal one. To determine the parameters for a wide range of supply voltages, the measurements of motor step responses were carried out for voltages from 5 V to 16 V. In the next step, the CPM-GWO method was applied to the obtained data and the motor parameters were determined. The obtained results are shown in Figure 11 (for a few selected supply voltages), in Figure 12 and in Table 2.

As can be seen in Figure 12 and Table 2, the values of almost all estimated parameters for different voltages are constant and they fluctuate around the average value. However, in the case of friction coefficient *b*, its value depends on the angular speed of the shaft. This problem with the friction coefficient is discussed further in the next section.

Similar results as presented in Table 2 can be also obtained using a single optimization calculation, using the measured parameters for different voltages to obtain a single set of parameters for the motor, valid for different voltages. Such techniques of parameter optimization have been recently proposed by the authors in [31].

## 4. Discussion

**Figure 13:**
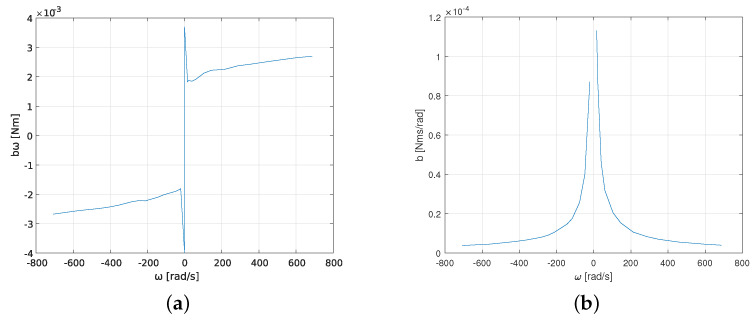
Friction torque and friction coefficient as functions of angular speed for the Buhler DC motor 31 × 51 1.13.021.764 obtained in experimental tests; (**a**) friction torque; (**b**) friction coefficient.

To confirm the dependence between friction coefficient *b* and angular speed ω, the friction characteristics of the motor as a function of its angular speed were measured. The measurement was carried out at the same test stand as explained in Section 3. The obtained results are shown in Figure 13b. The obtained friction force moment bω (Figure 13a,b) corresponds to the Stribeck curve [32], which is characteristic for DC motors [33]. For lower rotational speeds (less than 200 rad/s) the dependency between the friction moment and angular speed is highly nonlinear. For higher rotational speeds (over 200 rad/s) the dependency is almost linear (both for positive and negative speeds). These observations agree with Stribeck friction theory, according to which at lower speeds dry friction (boundary lubrication) is present, and for higher rotational speeds viscous friction (hydrodynamic lubrication) takes advantage. In the viscous friction area, the viscous friction coefficient is usually assumed to be constant, and these almost constant values of *b* can be observed in Figure 13b for rotational speeds greater than 200 rad/s. For lower speeds (less than 200 rad/s) the relationship between *b* and ω is highly nonlinear, because, in this speed range, dry friction takes advantage. Thus, the obtained results confirm that the commonly assumed linear viscous friction model is not correct for all rotational speed ranges, and Stribeck or other more accurate friction models [6,7] should be included.

**Figure 14:**
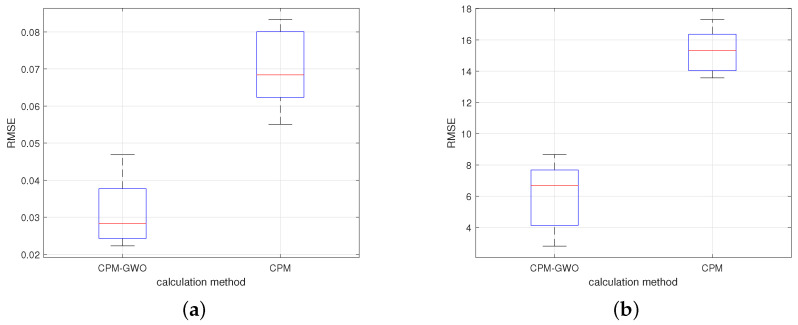
RMSE for two tested methods: CPM and CPM-GWO; (**a**) current; (**b**) angular speed.

To compare the results of the two methods, the root mean square error (RMSE) qualitative indicator was proposed:

(25) $$RMSE=\sqrt{\frac{1}{N}\sum_{i=1}^{N}{\left(p\left(i\right)-\hat{p}\left(i\right)\right)}^{2}}$$

where *p* is the step response sample for the parameters calculated with a given method, $\hat{p}$ is the experimental step response sample, *N* is the number of the step response samples. The indicator has been calculated for measurement data obtained from step responses at supply voltages ranging from 5 to 16 V. The more accurately the step response is reproduced, the smaller the indicator. The value of the RMSE for the current and speed is shown in Figure 14. As can be seen, the application of the CPM-GWO method reduces the error by about 50% when compared to the CPM method.

As shown in Figure 5 the GWO method used solely has great difficulties in estimating parameter values of the PMDC model. The GWO achieves significantly worse accuracy than the CPM-GWO, and stops improving just after the 15th iteration.

## 5. Conclusions

In the current paper, the CPM-GWO method has been proposed and evaluated in experimental tests. It has been demonstrated that the proposed method offers significantly lower matching error when compared to the CPM and GWO methods used solely. Moreover, the proposed method provides much lower computational time required to estimate the values of model parameters when compared to the GWO method.

The GWO method requires a priori information to provide correct and accurate estimates of the parameters. In the CPM-GWO method, this information comes from the initial estimates of the parameters achieved by the CPM, making the proposed method present correct, accurate, and reliable estimates.

The manufacturer provides values of nominal parameters that average the characteristics of the electric motors produced. Differences in the parameter values obtained using the presented CPM-GWO method in relation to the values declared by the manufacturer may result from differences arising at the production stage, wear of motor components (e.g., brushes, bearings).

The conducted tests have shown that friction coefficient *b* depends on the angular speed ω of the motor, and that this relation is in good agreement with Stribeck friction theory. Due to the observed variability of parameter *b*, it is worth including a more accurate friction model in future research related to this subject. In addition, future research could focus on more complex applications, such as when the motor is running under a constant or variable load.

## Figures and Tables

**Table 1:** Parameters of the Buhler DC motor 31 × 51 1.13.021.764 model obtained by the CPM, GWO, CPM-GWO methods and declared by the manufacturer for the nominal voltage of 12 V [30]. n/a—not applicable.

Parameter	Symbol		CPM	GWO	CPM-GWO	Reference	Unit
Resistance	*R*	avg	4.58	4.83	4.86	4.40	Ω
		σ	-	0.08	0.03	n/a	
		min	-	4.35	4.83	n/a	
		max	-	5.17	5.02	n/a	
Inductance	*L*	avg	3.40 × 10${}^{-3}$	4.82 × 10${}^{-3}$	4.78 × 10${}^{-3}$	6.16 × 10${}^{-3}$	H
		σ	-	9.24 × 10${}^{-5}$	8.63 × 10${}^{-5}$	n/a	
		min	-	4.40 × 10${}^{-3}$	4.36 × 10${}^{-3}$	n/a	
		max	-	4.99 × 10${}^{-3}$	4.92 × 10${}^{-3}$	n/a	
Electromotive constant	*K*	avg	2.44 × 10${}^{-2}$	6.54	2.49 × 10${}^{-2}$	2.50 × 10${}^{-2}$	$\frac{\mathrm{Vs}}{\mathrm{rad}}$
		σ	-	2.31	6.04 × 10^−5^	n/a	
		min	-	0.07	2.47 × 10${}^{-2}$	n/a	
		max	-	9.73	2.51 × 10${}^{-2}$	n/a	
Rotor inertia	*J*	avg	1.61 × 10${}^{-6}$	0.13	1.60 × 10${}^{-6}$	1.60 × 10${}^{-6}$	${\mathrm{kgm}}^{2}$
		σ	-	0.07	2.70 × 10${}^{-8}$	n/a	
		min	-	1.13 × 10${}^{-5}$	1.58 × 10${}^{-6}$	n/a	
		max	-	0.24	1.74 × 10${}^{-6}$	n/a	
Friction coefficient	*b*	avg	7.56 × 10${}^{-6}$	0.51	6.11 × 10${}^{-6}$	6.00 × 10${}^{-6}$	Nms/rad
		σ	-	0.29	7.81 × 10${}^{-7}$	n/a	
		min	-	2.25 × 10${}^{-7}$	2.16 × 10${}^{-6}$	n/a	
		max	-	0.99	6.64 × 10${}^{-6}$	n/a	

**Table 2:** Parameter values estimated by the proposed CPM-GWO method of the Buhler DC motor 31 × 51 1.13.021.764 for various voltages.

Source [V]	L [H]	K $\left[\frac{\mathrm{Vs}}{\mathrm{rad}}\right]$	J $\left[{\mathrm{kgm}}^{2}\right]$	R [Ω]	b [Nms/rad]
5	4.99 × 10${}^{-3}$	23.71 × 10${}^{-3}$	1.49 × 10${}^{-6}$	5.18	1.35 × 10${}^{-6}$
6	5.21 × 10${}^{-3}$	25.78 × 10${}^{-3}$	1.67 × 10${}^{-6}$	5.00	11.70 × 10${}^{-6}$
7	5.27 × 10${}^{-3}$	24.40 × 10${}^{-3}$	1.55 × 10${}^{-6}$	5.21	10.60 × 10${}^{-6}$
8	4.98 × 10${}^{-3}$	24.72 × 10${}^{-3}$	1.59 × 10${}^{-6}$	5.04	9.87 × 10${}^{-6}$
9	5.72 × 10${}^{-3}$	24.37 × 10${}^{-3}$	1.51 × 10${}^{-6}$	5.11	9.74 × 10${}^{-6}$
10	4.35 × 10${}^{-3}$	24.95 × 10${}^{-3}$	1.58 × 10${}^{-6}$	4.77	7.10 × 10${}^{-6}$
11	4.88 × 10${}^{-3}$	24.52 × 10${}^{-3}$	1.49 × 10${}^{-6}$	5.01	7.94 × 10${}^{-6}$
12	4.78 × 10${}^{-3}$	24.83 × 10${}^{-3}$	1.59 × 10${}^{-6}$	4.85	6.41 × 10${}^{-6}$
13	5.12 × 10${}^{-3}$	24.53 × 10${}^{-3}$	1.50 × 10${}^{-6}$	5.09	7.32 × 10${}^{-6}$
14	5.13 × 10${}^{-3}$	24.71 × 10${}^{-3}$	1.57 × 10${}^{-6}$	4.66	7.29 × 10${}^{-6}$
15	4.66 × 10${}^{-3}$	24.40 × 10${}^{-3}$	1.50 × 10${}^{-6}$	5.02	5.78 × 10${}^{-6}$
16	4.92 × 10${}^{-3}$	24.98 × 10${}^{-3}$	1.57 × 10${}^{-6}$	4.69	5.18 × 10${}^{-6}$
avg	5.00 × 10${}^{-3}$	24.66 × 10${}^{-3}$	1.55 × 10${}^{-6}$	4.97	8.53 × 10${}^{-6}$
** σ **	0.324 × 10${}^{-3}$	0.47 × 10${}^{-3}$	0.0529 × 10${}^{-6}$	0.18	2.43 × 10${}^{-6}$

## Data Availability

The data presented in this study are available on request from the corresponding author. The data are not publicly available due to the internal restrictions of the Bialystok University of Technology.
